# SNP markers tightly linked to root knot nematode resistance in grapevine (*Vitis cinerea*) identified by a genotyping-by-sequencing approach followed by Sequenom MassARRAY validation

**DOI:** 10.1371/journal.pone.0193121

**Published:** 2018-02-20

**Authors:** Harley M. Smith, Brady P. Smith, Norma B. Morales, Sam Moskwa, Peter R. Clingeleffer, Mark R. Thomas

**Affiliations:** 1 CSIRO Agriculture and Food, Glen Osmond, South Australia, Australia; 2 CSIRO Information Management & Technology, Clayton South, Victoria, Australia; Universidade de Lisboa Instituto Superior de Agronomia, PORTUGAL

## Abstract

Plant parasitic nematodes, including root knot nematode *Meloidogyne* species, cause extensive damage to agriculture and horticultural crops. As *Vitis vinifera* cultivars are susceptible to root knot nematode parasitism, rootstocks resistant to these soil pests provide a sustainable approach to maintain grapevine production. Currently, most of the commercially available root knot nematode resistant rootstocks are highly vigorous and take up excess potassium, which reduces wine quality. As a result, there is a pressing need to breed new root knot nematode resistant rootstocks, which have no impact on wine quality. To develop molecular markers that predict root knot nematode resistance for marker assisted breeding, a genetic approach was employed to identify a root knot nematode resistance locus in grapevine. To this end, a *Meloidogyne javanica* resistant *Vitis cinerea* accession was crossed to a susceptible *Vitis vinifera* cultivar Riesling and results from screening the F_1_ individuals support a model that root knot nematode resistance, is conferred by a single dominant allele, referred as *MELOIDOGYNE JAVANICA RESISTANCE1 (MJR1)*. Further, *MJR1* resistance appears to be mediated by a hypersensitive response that occurs in the root apical meristem. Single nucleotide polymorphisms (SNPs) were identified using genotyping-by-sequencing and results from association and genetic mapping identified the *MJR1* locus, which is located on chromosome 18 in the *Vitis cinerea* accession. Validation of the SNPs linked to the *MJR1* locus using a Sequenom MassARRAY platform found that only 50% could be validated. The validated SNPs that flank and co-segregate with the *MJR1* locus can be used for marker-assisted selection for *Meloidogyne javanica* resistance in grapevine.

## Introduction

Plant parasitic nematodes are major soil-borne pests that cause extensive damage to a wide range of crops with an estimated cost of $80 billion (USD) per year [1]. To date, greater than 4100 species of plant parasitic nematodes have been identified and classified as endoparasites (sedentary and migratory), semi-endoparasites and ectoparasites. In many horticultural crops including grapevine, rootstocks resistant to plant parasitic nematodes as well as other pests provide a sustainable approach to limit crop losses [2].

Root knot nematodes are sedentary endoparasites that cause extensive damage to a wide range of crop species including *V*. *vinifera* [3–5]. Root knot nematodes are typically found in sandy soils, and complete their life cycle by parasitizing roots of susceptible plants. The life cycle of the root knot nematode begins after the eggs hatch and release the free-living juvenile nematodes, which migrate through the soil. The juvenile nematode enters the root and moves intracellularly toward the root meristem then turns and migrates up the vascular cylinder. In the zone of differentiation, the juvenile nematode establishes a permanent feeding site by secreting effectors that function to induce the formation of multinucleate “giant cells”. Subsequently, cells surrounding the giant cells undergo cell division and cell expansion until a gall is formed. Root knot nematodes feed on the cytoplasm of the giant cells through their stylets. After undergoing several molts, the nematode develops into an egg-laying female and the life cycle is completed [3–5].

In Mediterranean climates throughout the world, *M*. *arenaria*, *M*. *incognita* and *M*. *javanica* are the three major root knot nematode species that parasitize the roots *V*. *vinifera* and susceptible *Vitis* rootstocks [2]. Experimental results show that extensive gall formation and nematode feeding ultimately impairs root function, leading to a reduction in shoot and root growth, as well as crop production in grapevine [4,6–8]. Moreover, root knot nematode feeding can also result in an increase in susceptibility to abiotic stresses as well as other pests and pathogens [4,7], which further decreases productivity. While the use of fumigants and nematicides are used as a means to control root knot nematode parasitism, these chemical treatments are often ineffective, expensive and damaging to the environment or not permitted [7]. In contrast, the use of rootstocks provides a viable and sustainable management tool to maintain vine productivity in the presence of root knot nematodes.

The commercially available grapevine rootstocks that display resistance to root knot nematode such as Dog Ridge (*V*. *x champinii*), Ramsey (*V*. *x champinii*), Freedom (1613–59 [1613 Couderc (*V*. *acerifolia* x Othello) x 3306 Couderc (*V*. *riparia* x *V*. *rupestris*)] x *V*. *x champinii*, open pollinated Dogridge 5) [9] and Harmony (1613–39 [1613 Couderc, open pollinated] x *V*. *x champinii*, open pollinated Dogridge 5) are highly vigorous and take up excess potassium, which reduces wine quality [10,11]. Therefore, an understanding of the genetic control of root knot nematode resistance is essential for breeding new rootstocks with resistance to these soil pests, as well as other horticultural favorable traits. Genetic analysis from interspecies crosses indicates that root knot nematode resistance is conferred by a single dominant allele in *V*. *champinii* and 1613 Couderc [12]. The inheritance of root knot nematode resistance was examined further in commercially available rootstocks, such as Dog Ridge, Ramsey, Freedom, Harmony, 1616 Couderc (*V*. *acerifolia* x *V*. *riparia*) and 1613 Couderc, using a Design II mating analysis [13]. Results from this study indicate that a single dominant allele confers root knot nematode resistance in these rootstocks. At this time it is unclear if the resistance allele is identical in rootstocks of different parentage. Experimental evidence indicates that a recessive resistant allele is also present in grapevine based on inheritance of root knot nematode resistance in *V*. *rupestris* x *V*. *vinifera* [12] and 1613 Couderc x 3309 Couderc (*V*. *riparia* x *V*. *rupestris*) [13]. Since the emergence of virulent root knot nematode pathotypes, which have broken resistance to Harmony, Freedom and Ramsey [14–17], recent breeding efforts for root knot nematode resistance have led to the production of five new rootstocks UCD-GRN1-5 in California [18].

Marker assisted selection is an effective approach to combine desired traits of interest into crop breeding programs [19–21]. Next generation sequencing technologies, such as genotyping-by-sequencing (GBS), provide a cost effective and efficient approach for single nucleotide polymorphism (SNP) discovery and mapping of desirable traits in *Vitis* spp. [22–29], as well as other perennial crops [30]. A previous study showed that *V*. *cinerea* Engelm. Ex Millard is highly resistant to a *M*. *incognita* pathotype identified in California [31]. It was concluded that this accession would be a valuable resource of root knot nematode resistance for rootstock breeding. Therefore, in the current study, we identified a *V*. *cinerea* accession called a C2-50, which provides complete resistance to an aggressive *M*. *javanica* pathotype. In order to develop molecular markers for predicting root knot nematode resistance in grapevine, the *V*. *cinerea* C2-50 accession was crossed to a susceptible *V*. *vinifera* cultivar Riesling. Using a root knot nematode-screening assay, F_1_ individuals were screened and results indicate that root knot nematode resistance is conferred by a single dominant allele. A genotyping-by-sequencing approach was used for association genetics and to generate genetic maps. Both approaches were used to localize the root knot nematode resistance locus to a chromosome region. A subset of SNPs surrounding the root knot nematode resistance locus was validated for potential use for marker-assisted breeding. Using an in vitro screening method, root knot nematode resistance appears to be mediated by cellular necrosis in the root meristem, which may function to limit migration of the nematode.

## Materials and methods

### Plant material

Ninety F_1_ individuals derived from a cross of a *V*. *cinerea* accession C2-50 (female parent) with *V*. *vinifera* Riesling (male parent) were planted at the CSIRO vineyard. DNA typing was performed using ten microsatellite markers, VVS2 [32], VVMD5, VVMD7 [33], VrZAG62, VrZAG79 [34], VVMD27, VVMD28, VVMD32, VVMD36 [35] and VVIP31 [36] to verify the parentage of the F_1_ individuals before initiating the root knot nematode screening assay. Three dormant cuttings for C2-50, Riesling and the 90 F_1_ individuals were propagated and transplanted into 6.5 x 6.5 x 20 cm pots for nematode screening. Four to five weeks after transplanting the rooted cuttings in soil, the vines were screened for root knot nematode resistance.

### Root knot nematode screening

The aggressive *M*. *javanica* ‘pt 1103P’ pathotype, which is able to effectively parasitize the moderately resistant 1103 Paulsen rootstock, was used in the root knot nematode screening assay [37]. *M*. *javanica* ‘pt. 1103P’ was propagated on susceptible *Solanum lycopersicum* (tomato) cv California Red Cherry plants at 22–25°C in a glasshouse. Eight weeks after inoculation, galls containing the *M*. *javanica* ‘pt. 1103P’ egg masses were dissected from the infected tomato roots and incubated in water at 30°C for 72 h to promote hatching. Subsequently, a 500 μl aliquot of hatched nematodes was pipetted onto a microscope slide and the number of juvenile nematodes was counted using a Zeiss Stemi 2000-C stereomicroscope. Based on the number of nematodes per 500 μl, approximately 1000 *M*. *javanica* ‘pt. 1103P’ juveniles were added to each potted cutting and incubated for 8 weeks in the glasshouse at 22–25°C. Plants were classified as susceptible if the root knot nematodes are able to complete their life cycle by producing egg masses on the roots. For egg mass determination, the roots for each replicated cutting were washed gently in water to remove the soil and incubated in 0.01 g/L of erioglaucine disodium salt for 60 min to stain the egg masses. Egg masses were counted under a Daylight Magnifying Lamp (Model number: A22020-01). A chi-square goodness of fit test was performed with the phenotype data using X^2^ = total of (O-E)^2^/E for resistance and susceptible phenotypes, with one degree of freedom. For each replicated screen, C2-50 and Riesling were included as controls for resistant and susceptible genotypes.

### Genotyping-by-sequencing

DNA isolation for C2-50, Riesling and the F_1_ individuals was performed at the Australian Genome Research Facility using the Nucleic Acid Extraction service, which utilizes the NucleoSpin^®^ 96 Plant II DNA extraction kit (http://www.mn-net.com). The Cornell University Biotechnology Resource Center (BRC) provided the GBS service as described by [38]. Briefly, genomic DNA was digested with the *Ape*KI methylation sensitive restriction endonuclease to reduce genome complexity prior to the construction of the library. Single-end 100 bp sequence reads were generated using Illumina HiSeq 2000/2500 (Illumina Inc., San Diego, CA, USA). Note: the library was generated from a 96 well-plate containing the 90 F_1_ individuals and two samples of Riesling and C2-50. This library was sequenced 2X in order to maximize the number of reads at each locus. SNP discovery and genotyping was performed by the BRC as described by [39]. In this procedure, the single-end 100 bp reads were processed to 64 bp sequence tags and aligned to the 12X *V*. *vinifera* ‘PN40024’ reference genome [40,41] using Burrows-Wheeler Aligner maximal exact match using default parameters [42]. SNPs were called using TASSEL-GBS pipeline, v3.0.166 [39]. The variant call format (VCF) output file [43] consisted of 509,293 SNPs that were present in >90% of the progeny and had a minor allele frequency >0.01. The called SNPs were filtered using VCFtools v.1.12b [43]. In this procedure, SNPs were filtered with an average depth of read coverage >10 and a minor allele frequency >0.2, 0% missing data and a genotype quality score >98%. Next, SNPs with an allele frequency between 40–60% were retained. After filtering, the SNP set was parsed into two data sets based on a pseudo-test cross mapping strategy [44]. The C2-50 SNP set containing 3974 SNPs was obtained by retrieving SNPs that were heterozygous in C2-50 and homozygous in Riesling. The Riesling SNP set consisted of SNPs that were homozygous in C2-50 and heterozygous in Riesling. The Riesling SNP set contains 2973 SNPs. The 18,124, C2-50 and Riesling SNP sets can be accessed at http://datadryad.org/review?doi=doi:10.5061/dryad.1d7n9.

### Single SNP association analysis

Single SNP association analysis was performed with the C2-50 (3974) and Riesling (2973) SNP sets to identify markers that associate with *M*. *javanica* ‘pt 1103P’ resistance using TASSEL 5.0 [45]. Results from the root knot nematode screening were converted to a ‘trait’ file in which the resistant and susceptible phenotypes were converted to a “0” or “1” numerical value, respectively. After removing C2-50 and Riesling from the SNP sets, the ‘intersect join’ command was used to join the ‘trait’ file with the C2-50 (3974) and Riesling (2973) SNP sets. This command produced the C2-50 and Riesling numerical data sets for the 90 F_1_ individuals. Association mapping using the general linear model (GLM) was used to analyze the C2-50 and Riesling numerical data sets with 1000 permutations. For association mapping using the mixed linear model (MLM) [46], a kinship file was produced from the C2-50 (3974) and Riesling SNP (2973) SNP sets using the kinship command with scaled identity by state (IBS). Next, MLM analysis was performed with the C2-50 and Riesling kinship and numerical data sets. Variance component estimation was performed with ‘no compression’ using the ‘population parameters previously determined (P3D)’ [47]. The raw *p*-values identified by GLM and MLM were adjusted for false discovery rate according to the Bonferroni and Benjamini-Hochberg procedures using the R/multtest package [48].

### Linkage map construction

Linkage mapping was performed with R/OneMap [49] using the Kosambi function. SNPs were ordered with a linkage LOD of 6.0 and a recombination frequency of 0.25. The C2-50 (3974 SNPs) and Riesling (2973 SNPs) SNP sets were reduced to 367 and 404 SNPs, respectively, by adjusting the distance between markers to 2.0–5.0 cM and removing markers with a high mean recombination fraction and low mean LOD score using the ‘rf.graph.table’ function. The C2-50 and Riesling SNP sets used for linkage mapping can be accessed at http://datadryad.org/review?doi=doi:10.5061/dryad.1d7n9.

### Interval mapping

Interval mapping was performed using R/QTL [50]. The C2-50 (367) and Riesling (403) genetic maps generated in R/OneMap were used in this analysis. In addition, interval mapping was performed with the 372 C2-50 SNP set, which contains 8 validated markers. To create this file, S18_30104122 and S18_33162606 were removed from the 367 C2-50 SNP set, as the genotype for these markers could not be verified. Next, only eight of the validated markers at the *MJR1* locus were used to create 372 C2-50 SNP set, S18_26580875 (92.5 cM), S18_26558715 (93.6 cM), S18_27884817 (95.9 cM), S18_30104225 (97.0 cM), S18_30236024 (98.1 cM), S18_31160355 (99.2 cM), S18_32680428 (100.3 cM) and S18_33954011 (101.4 cM). Note: S18_27884817 was present in the 367 SNP set. To perform the binary trait method, the resistant and susceptible phenotypes were converted to values equal to 0 and 1, respectively. The one-dimensional genome scan was performed using the *scanone* function with the argument *model = ‘binary’*. The LOD threshold value was determined by 1000 permutations with *alpha = 0*.*05*. For single-QTL analysis, two phenotype data sets were used for interval mapping: (1) the average number of egg masses per root system (EM/R) and (2) the average number of egg masses per root weight (g: EM/RW). After importing the C2-50 and Riesling maps together with the genotype and phenotype data, the *calc*.*genoprob* function with *step = 1* was used to calculate multipoint genotype probabilities. Next, the one-dimensional genome scan was performed using the *scanone* function with the Haley-Knott regression method, *method = “hk”*. LOD threshold values were estimated by 1000 permutations (*alpha = 0*.*05*).

### SNP validation

DNA was isolated from 65 F_1_ individuals plus C2-50 and Riesling Nucleic Acid using the NucleoSpin^®^ 96 Plant II DNA extraction kit. Thirty SNPs that spanned the *M*. *javanica* ‘pt. 1103P’ resistance locus were validated using the SNP genotyping Sequenom MassARRAY iPLEX platform (Sequenom, San Diego, CA, USA) [51]. This SNP genotyping platform service was provided by the Australian Genome Research Facility (http://www.agrf.org.au/services/genotyping). The SNP genotypes determined by the GBS pipeline and the Sequenom MassARRAY platform were compared to validate each SNP.

### In vitro root knot nematode assay

To obtain a sterile population of *M*. *javanica* ‘pt 1103P’, twenty egg masses were isolated from tomato roots and surface sterilized by vigorously shaking the eggs in 0.5% chlorine solution for 4 min at 22°C. After centrifugation at 1000 xg for 5 min, the chlorine solution was removed and the eggs were washed with sterile water five times in laminar flow cabinet to remove residual chlorine. After the final wash, the eggs were resuspended in 1.0 ml of sterile water and incubated at 30°C to promote hatching. To maintain a sterile culture of root knot nematodes, *Cucumis sativus* roots were inoculated with sterile *M*. *javanica* ‘pt 1103P’ juveniles and egg masses were collected and hatched [52].

Single node cuttings, 3–4 cm in length with a stem diameter of approximately 5 mm, were isolated from developing shoots of C2-50, Riesling and 12 C2-50 x Riesling F_1_ individuals. After removing the leaves, the nodal cuttings were washed in sterile water. Ten nodes from each genotype were incubated in a solution containing 5% active chlorine plus 0.1% Tween 20 for 12 hrs at 22°C. In a laminar flow cabinet, nodal cuttings were rinsed three times with sterile water and the basal end of the node was trimmed and inserted in callus initiation media (PIV) [53]. After bud initiation, 20–30 mm shoots were removed and placed in root initiation medium (RIM) [54].

For the root knot nematode screening, 8–10 roots were excised from each genotype and placed on nematode screening media (NSM) containing macroelements and microelements [55], B5 vitamins [56], FeEDTA (7.44 g/L Na_2_EDTA and 1.86 g/L FeSO_4_), 3% sucrose and 0.8% phytagel, pH 5.7 (KOH), for two days at 24°C in the dark. After isolating *M*. *javanica* ‘pt 1103P’ juveniles as described by [52], the number of nematodes per 1.0 ml sample was determined by pipetting a 25–50 μl onto a microscope slide and counting the number of nematodes under a Zeiss Stemi 2000-C stereomicroscope. Using this method, we estimated the number of *M*. *javanica* ‘pt 1103P’ juveniles per volume of sample. Roots from C2-50, Riesling and 12 C2-50 x Riesling F_1_ individuals were inoculated with approximately 25 *M*. *javanica* ‘pt 1103P’ juveniles and incubated at 24°C in the dark. Roots were visually inspected for a hypersensitive response (HR), root growth cessation and gall formation every 10–12 hours. For each genotype, control roots were inoculated with sterile water to access the viability of the roots. For each experiment, not all of the roots on each plate displayed a response, such as gall formation or HR, after incubation with the juvenile nematodes. Therefore, we calculated the percentage of roots that displayed gall formation or HR. Roots from each genotype were screened at least three times. Roots were imaged using a Zeiss Stemi 2000-C stereomicroscope with a Spot FLEX^®^ digital camera.

To visualize nematodes, the in vitro grown roots were incubated in 10% bleach for 5–10 min, three days after inoculation with *M*. *javanica* pt ‘1103P’. During this step, roots were monitored during the incubation period and immediately placed in water when the transparency of the necrotic region was reduced. After rising 5 times in water, the roots were boiled in an acid fuchsin staining solution (3.5% acid fuchsin and 25% acetic acid in water) for 10 sec then cooled to 22°C. Finally, the roots were incubated in a destaining solution (33% acetic acid and 33% glycerol in water) for 4 hours and the roots were imaged using a Zeiss Axioskop2 microscope with a Spot FLEX^®^ digital camera.

## Results

### Phenotype analysis of root knot nematode resistance

Three propagated cuttings from *V*. *cinerea* C2-50, an accession in the CSIRO Rootstock Collection, were screened for root knot nematode resistance and results showed that *M*. *javanica* ‘pt 1103P’ failed to parasitize this accession, as no egg mass development occurred on the roots for each of the replicated cuttings (Table 1). Therefore, C2-50 was chosen as the female parent for mapping a root knot nematode resistance locus in grapevine. As indicated by the mean egg masses and mean egg masses per root weight, *M*. *javanica* ‘pt. 1103P’ was able to effectively parasitize the roots of the Riesling demonstrating that this *V*. *vinifera* cultivar is susceptible to *M*. *javanica* ‘pt. 1103P’ (Table 1). Therefore, to map *M*. *javanica* ‘pt. 1103P’ resistance, a cross was made between C2-50 and Riesling.

**Table 1:** *M*. *javanica* ‘pt. 1103P’ resistance in the C2-50 and Riesling.

Genotype	EM	DR-wt	EM/DR-wt
C2-50	0	2.89 ±0.94	0
Riesling	20.0 ±15.6	2.90 ±0.75	5.58 ±2.04

EM = mean egg masses; DR-wt = mean dry root weight

EM/DR-wt = mean egg masses per dry root weight (g)

Using a glasshouse based root knot nematode screening method [37], 90 F_1_ individuals were screened for *M*. *javanica* ‘pt. 1103P’ resistance. In this screening assay, three propagated cuttings per individual were screened. Results showed that *M*. *javanica* ‘pt 1103P’ was able to effectively parasitize the roots for 39 out of the 90 F_1_ individuals. In this experiment, gall and egg mass development occurred in all three replicates for each of the 39 susceptible genotypes. The average number of egg masses per root system for these susceptible F_1_ individuals was 34.7. For the remaining 51 F_1_ individuals screened for *M*. *javanica* ‘pt. 1103P’ resistance, no egg masses or galls were detected on the roots in all three replicates. The distribution of the phenotype data is displayed in S1 Fig. A chi-square goodness of fit test was performed to determine whether the phenotypic ratio for *M*. *javanica* ‘pt. 1103P’ resistance segregates with a 1:1 ratio. Using one degree of freedom, the chi-square value was 1.6, which is less than the critical value of 3.84. In addition, the probability was >0.05 indicating that *M*. *javanica* ‘pt. 1103P’ resistance segregates with a 1:1 ratio. Therefore, the data support a model that *M*. *javanica* ‘pt. 1103P’ resistance, referred to as *MELOIDOGYNE JAVANICA RESISTANCE1 (MJR1)*, is conferred by a single allele in C2-50, which can be explained by two hypotheses. In the first hypothesis, C2-50 is heterozygous dominant for *MJR1* (*MJR1/mjr1*) and Riesling is homozygous recessive (*mjr1/mjr1*). Alternatively, the second hypothesis predicts that resistance is conferred by a recessive allele (*mjr1*), in which C2-50 is homozygous (*mjr1/mjr1*) and Riesling is heterozygous (*MJR1/mjr1*) for the recessive allele.

### Genotyping-by-sequencing and SNP filtering

**Fig 1:**
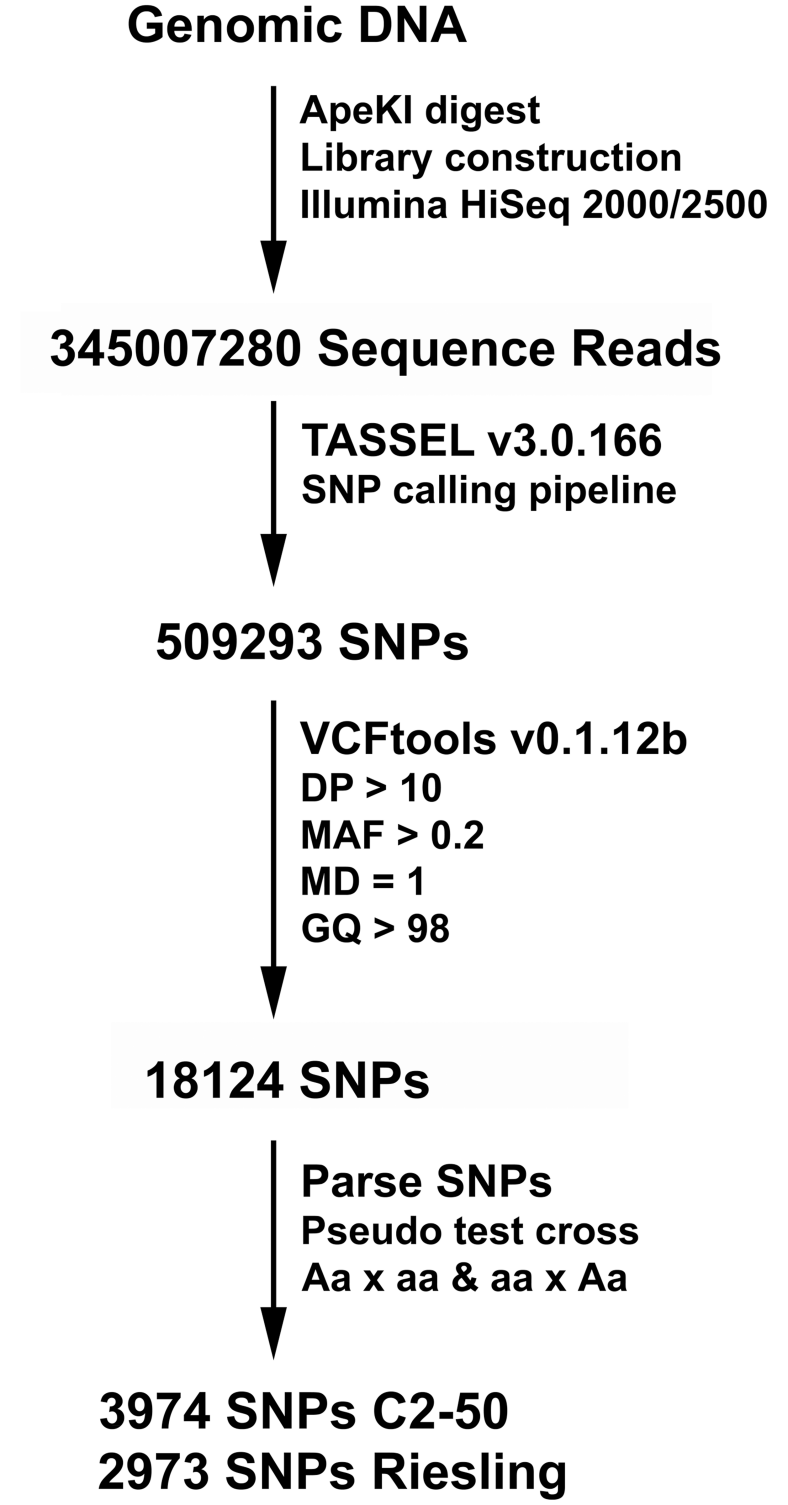
Flow chart for GBS and filtering of the C2-50 and Riesling SNP sets. Genomic DNA was isolated from the parents and F_1_ progeny genotypes. Genome complexity was reduced by digesting the genomic DNA with the *Ape*KI methylation sensitive restriction endonuclease. Libraries were sequenced using Illumina HiSeq 2000/2500 and sequence reads were aligned to the PN40024 reference genome [40,41] using BWA [42]. After using the TASSEL V3.0.166 SNP calling pipeline [39], a 509,293 SNP set was generated. The called SNPs were filtered using VCFtools [43] with a depth of read (DP) > 10, minor allele frequency (MAF) > 0.2, missing data (MD) = 1, and a genotype quality score (GQ) > 98. This filtering step reduced the SNP set to 18,124. SNPs were parsed using a pseudo test cross strategy [44]. The C2-50 and Riesling SNP sets contained 3974 and 2973 SNPs, respectively.

**Fig 2:**
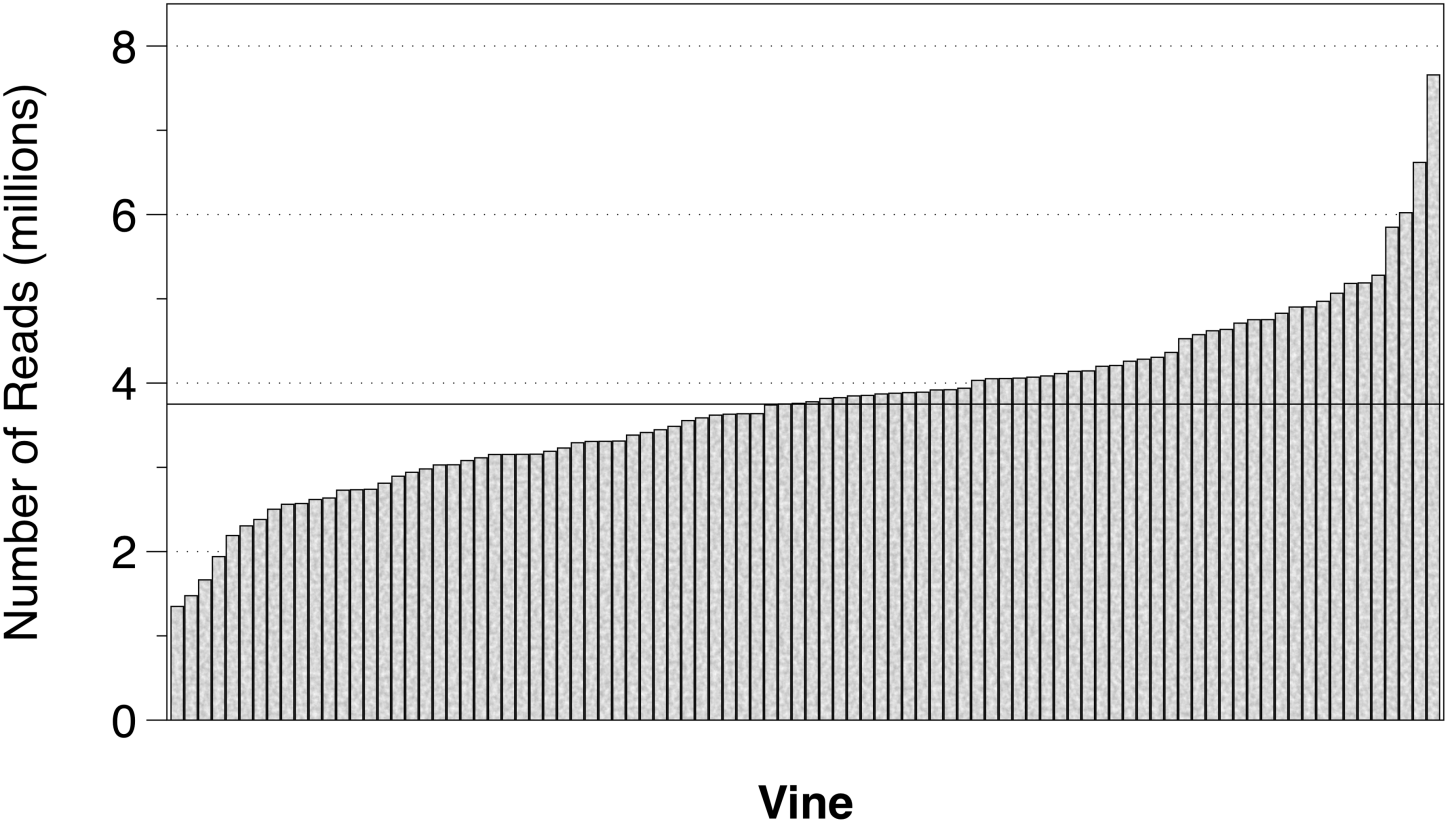
The number of sequence reads per vine. The number of sequence reads produced for the 90 F_1_ individuals and two parents are displayed. Vine and number of reads (millions) are on the x- and y-axis, respectively. The number of reads ranged from 1,348,872 to 6,021,751 per vine. The number of sequence reads obtained for C2-50 and Riesling were 7,657,007 and 6,618,727, respectively. The *horizontal line* indicates the average number of sequence reads.

Genotyping-by-sequencing (GBS) was performed on the 90 F_1_ individuals, as well as C2-50 and Riesling parents. Next generation sequencing produced 345,007,280 acceptable sequence reads (Fig 1) and the average number of sequenced reads per vine was 3,750,079.13. The distribution of sequence reads ranged from 1,348,872 to 7,657,007 (Fig 2). After SNP calling, a SNP set containing 509,293 markers was produced (Fig 1). Given that grapevine is highly heterozygous, SNPs were filtered with an average depth of sequence coverage greater than 10 and a genotype quality score greater than 98%, to reduce genotyping errors (Fig 1). After SNP filtering, the 509,293 SNP set was reduced to 18,124 SNPs (Fig 1). In order to perform single SNP association and genetic mapping, a pseudo-testcross mapping approach was used [44], and this reduced the 18,124 SNP set, producing the C2-50 and Riesling SNP sets containing of 3974 and 2973 markers, respectively (Fig 1). Note: SNPs heterozygous in C2-50 and homozygous in Riesling were retained in the C2-50 SNP set while the opposite set of SNPs was retained in the Riesling SNP set.

### Single SNP association analysis

A single SNP association analysis using GLM was performed to map *MJR1* using the C2-50 (3974 SNPs) and Riesling (2973 SNPs) SNP sets. Using this approach, 7 SNPs on chromosome 18 from position 31787750 to 33070986 in the C2-50 SNP set had high association based on *p*-values equal to 0 and R^2^ equal to 1 (Table 2). In addition, most of the immediate set of flanking markers from position 30104122 to 31481177 and 33162605 to 34140592 also displayed high association with *p*-values ranging from 4.26E-58 to 1.45E-27 and R^2^ between 0.789 to 0.955 (Table 2). In addition, SNPs with significant association extended beyond positions 33162605 and 34140592 (S1 Table). To further evaluate SNP association for root knot nematode resistance, a mixed linear model (MLM) approach was used and results showed that 19 SNPs from position 30226628 to 33276771 had *p*-values ranging from 1.43E-06 to 4.23E-12 and R^2^ between 0.496 to 0.871 (S2 Table). In contrast to C2-50, markers from the Riesling SNP set failed to show significant association using single SNP association with GLM or MLM (S3 and S4 Tables). Taken together, single SNP association results indicate that *MJR1* is located on chromosome 18 in C2-50. Given *M*. *javanica* ‘pt1103P’ resistance only associates with markers from the C2-50 SNP set supports the hypothesis that *MJR1* is conferred by a single dominant allele, which is heterozygous in C2-50 (*MJR1/mjr1*).

**Table 2:** Single SNP association (GLM) statistics for *M*. *javanica* ‘pt1103P’ resistance.

SNP ID	Chr	Position	Raw *p*-value	Adj. *p*-value Bonferroni	Adj. *p*-value Ben Hoch	R^2^
S18_30104122	18	30104122	1.18E-34	4.69E-31	9.57E-33	0.825
S18_30104225	18	30104225	5.32E-40	2.11E-36	5.56E-38	0.868
S18_30226628	18	30226628	2.54E-47	1.01E-43	3.26E-45	0.910
S18_30235933	18	30235933	3.66E-31	1.45E-27	2.85E-29	0.789
S18_30236024	18	30236024	2.54E-47	1.01E-43	3.26E-45	0.910
S18_30381428	18	30381428	2.36E-60	9.38E-57	4.26E-58	0.955
S18_30515641	18	30515641	2.54E-47	1.01E-43	3.26E-45	0.910
S18_30711041	18	30711041	2.36E-60	9.38E-57	4.26E-58	0.955
S18_30722375	18	30722375	2.36E-60	9.38E-57	4.26E-58	0.955
S18_30764625	18	30764625	2.36E-60	9.38E-57	4.26E-58	0.955
S18_31010973	18	31010973	5.80E-35	2.30E-31	4.80E-33	0.828
S18_31059094	18	31059094	2.36E-60	9.38E-57	4.26E-58	0.955
S18_31158466	18	31158466	0.034	1	0.251	0.051
S18_31158467	18	31158467	0.034	1	0.251	0.051
S18_31160355	18	31160355	2.36E-60	9.38E-57	4.26E-58	0.955
S18_31164496	18	31164496	2.36E-60	9.38E-57	4.26E-58	0.955
S18_31196588	18	31196588	2.36E-60	9.38E-57	4.26E-58	0.955
S18_31481177	18	31481177	2.36E-60	9.38E-57	4.26E-58	0.955
S18_31547619	18	31547619	0.608	1	0.922	0.003
S18_31624788	18	31624788	0.450	1	0.911	0.007
S18_31787750	18	31787750	0	0	0	1
S18_31822250	18	31822250	7.49E-48	2.98E-44	1.24E-45	0.913
S18_31886894	18	31886894	0	0	0	1
S18_32027399	18	32027399	1.77E-60	7.03E-57	4.26E-58	0.955
S18_32680428	18	32680428	0	0	0	1
S18_33070954	18	33070954	0	0	0	1
S18_33070972	18	33070972	0	0	0	1
S18_33070983	18	33070983	0	0	0	1
S18_33070986	18	33070986	0	0	0	1
S18_33162605	18	33162605	2.36E-60	9.38E-57	4.26E-58	0.955
S18_33162606	18	33162606	5.32E-40	2.11E-36	5.56E-38	0.868
S18_33276771	18	33276771	2.36E-60	9.38E-57	4.26E-58	0.955
S18_33388900	18	33388900	5.80E-35	2.30E-31	4.80E-33	0.828
S18_33757536	18	33757536	0.977	1	0.978	0.000
S18_33876484	18	33876484	2.54E-47	1.01E-43	3.26E-45	0.910
S18_33876485	18	33876485	2.54E-47	1.01E-43	3.26E-45	0.910
S18_33954011	18	33954011	2.36E-60	9.38E-57	4.26E-58	0.955
S18_33959722	18	33959722	5.32E-40	2.11E-36	5.56E-38	0.868
S18_34060245	18	34060245	5.80E-35	2.30E-31	4.80E-33	0.828
S18_34140592	18	34140592	1.31E-47	5.21E-44	2.08E-45	0.912

Raw *p*-values obtained from GLM were adjusted (Adj.) using Bonferroni and Benjamini-Hockberg (Ben Hoch). Note: this table only contains single SNP association results for a subset of SNPs on chromosome 18 (Chr 18) from the C2-50 (3974 SNP set). Position refers to the location of the SNP in the PN40024 reference genome. Results for single SNP association with the entire C2-50 (3974 SNPs) and Riesling (2973 SNPs) SNP sets is shown in S1 and S2 Tables, respectively.

### Genetic mapping of the *MJR1* locus

The C2-50 and Riesling SNP sets were reduced to 367 and 403 SNPs, respectively, as described in the material methods and R/OneMap was used to curate and construct the genetic maps. For each SNP set, 19 linkage groups (LGs) were produced and the final size for the C2-50 and Riesling genetic maps were 1587.3 and 1706.4 cM, respectively (S5 and S6 Tables), which is similar in size to other *Vitis* genetic maps produced by next generation sequencing [23,25,26,28]. The map density or average distance between SNP markers for C2-50 and Riesling genetic maps was 4.3 and 4.2 cM, respectively.

**Fig 3:**
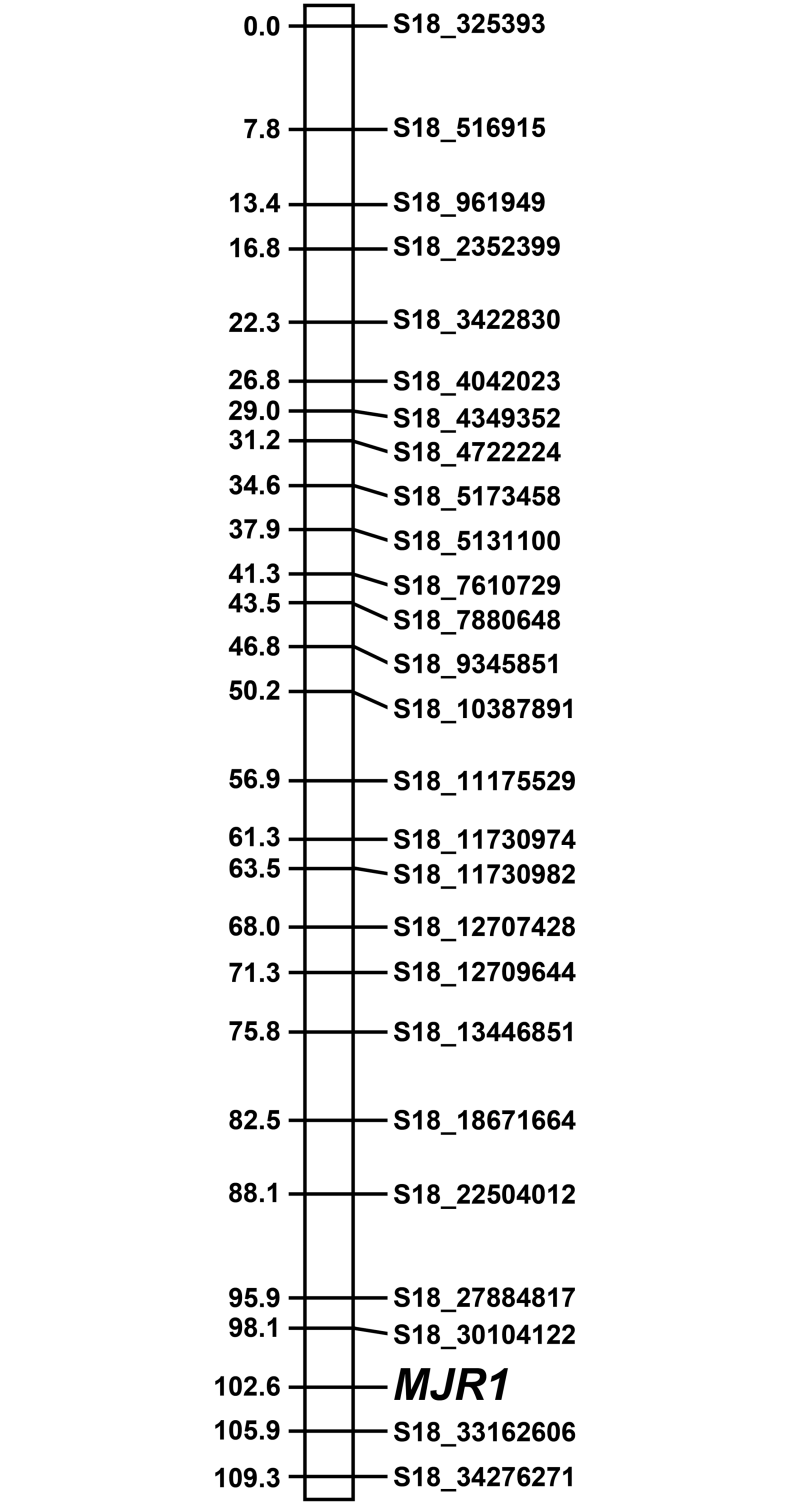
Linkage mapping of *MJR1* maps on LG18 at 102.6 in C2-50. The order of the markers on LG18 was determine using the 367 SNP set plus the *MJR1* marker followed by genetic mapping using R/OneMap. SNPs designated on the right side of linkage group. Genetic distance in cM is displayed on the left side of the linkage group. Note: all SNPs on LG18 were located on chromosome 18 in the PN40024 reference genome, as indicated by the position number provided in the marker name.

In order to map the *MJR1* locus, the phenotype data for each F_1_ individual was converted to a genotype. Given that the phenotype data closely matched a 1:1 ratio and SNPs from C2-50 on chromosome 18 display high association with *M*. *javanica* ‘pt 1103’ resistance, it is highly likely that the genotype of the C2-50 was heterozygous for *MJR1*, while Riesling was homozygous recessive. Therefore, resistant and susceptible F_1_ individuals were assigned either an *MJR1/mjr1* or *mjr1/mjr1* genotype, respectively. To map the *MJR1* locus, the *MJR1* marker was included in the reduced C2-50 (367) and Riesling (403) SNP sets and linkage analysis was performed using R/OneMap. Results from the genetic mapping showed that *MJR1* mapped to linkage group 18 (LG18) at 102.6 cM using the C2-50 367 SNP set (Fig 3). In this analysis, *MJR1* was flanked by S18_30104122 and S18_33162606 at 98.1 and 105.9 cM, respectively (Fig 3). When the *MJR1* marker was included in the Riesling 404 SNP set, *M*. *javanica* ‘pt 1103P’ resistance was not mapped to any of the 19 linkage groups (data not shown).

### Interval mapping of *MJR1*

**Fig 4:**
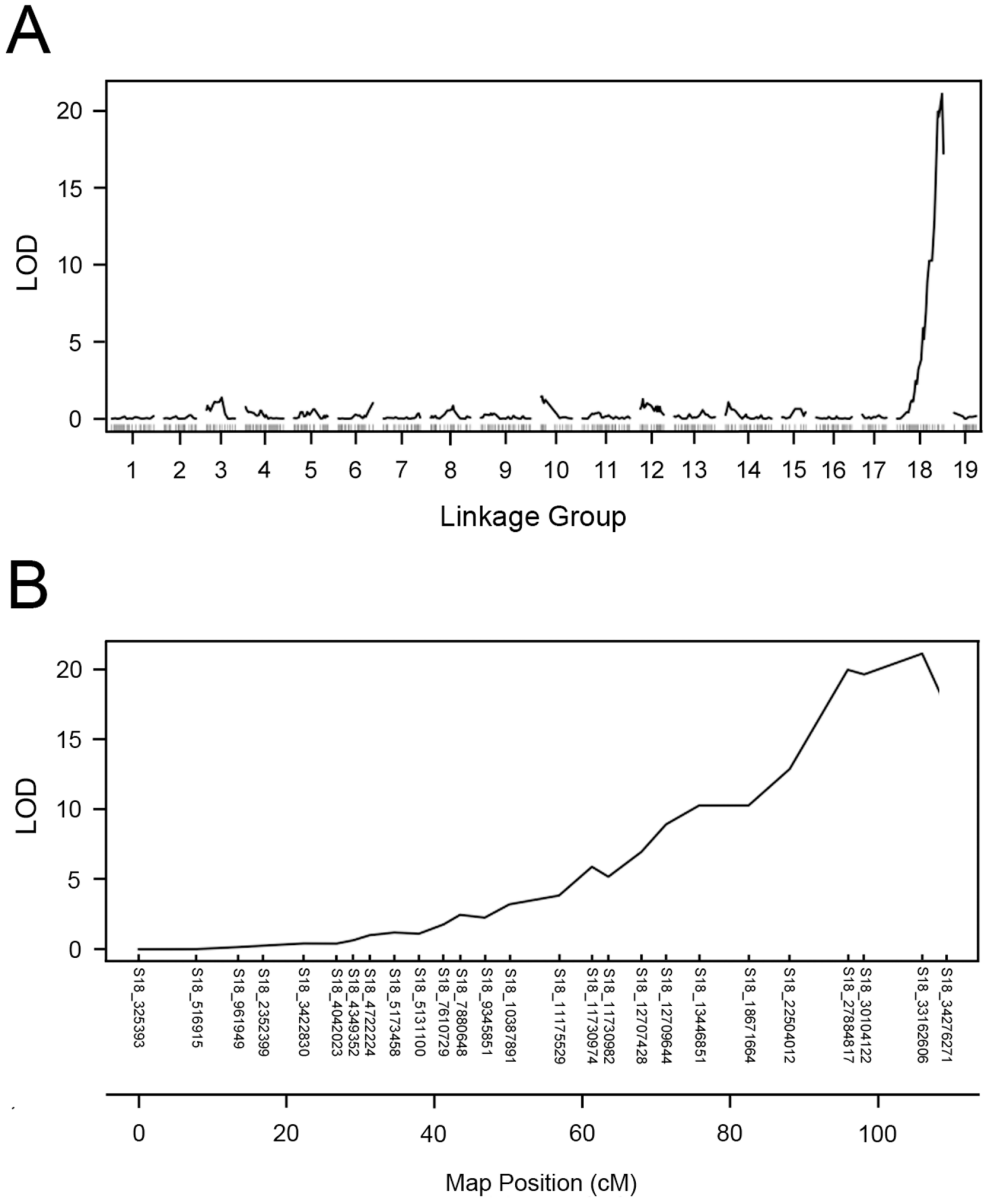
Interval mapping using the binary model for *MJR1*. (A) A single LOD peak on LG18 reached a maximum of 21.1 in C2-50. Linkage group number and LOD scores are displayed on the x-axis and y-axis, respectively. (B) A diagram of LG18 displayed the LOD maximum peak at 106 cM. The SNPs and map position (cM) are shown on the x-axis and LOD scores on the y-axis. The threshold, as determined by 1000 permutations, was 3.02.

Interval mapping was used to further localize *MJR1* using the binary and standard models available in R/QTL [50]. Similar to linkage mapping performed with R/OneMap, a binary trait was created in which resistant and susceptible individuals were assigned a value equal to 0 and 1, respectively. The binary mapping results showed that a single LOD peak was detected on LG18 (Fig 4A). This peak had a LOD maximum score of 21.1 (*p*-value = 0.0) at 106 cM, which is in close proximity to S18_33162606 at 105.9 cM (Fig 4B). The LOD score of 21.1 is above the threshold value of 3.02. Markers above the LOD threshold were not detected when the binary method was used to map *MJR1* with the Riesling 403 SNP set (S2 Fig).

Using the standard model of interval mapping, *M*. *javanica* ‘pt 1103P’ resistance localized to a single QTL on LG18 in the C2-50 367 SNP set (S3A and S3B Fig). Using the *M*. *javanica* resistance trait, EM/R (see Materials and methods), *MJR1* mapped at 97 cM, with a LOD score equal to 7.27 (*p*-value = 0.003) (S3A Fig). Alternatively, using the EM/RW trait (see Materials and methods), results showed that *M*. *javanica* resistance mapped to position 98 cM with a LOD score of 6.8 (*p*-value = 0.008) (S3B Fig). Both LOD scores derived for the *M*. *javanica* ‘pt 1103P’ resistance QTL were above the LOD threshold values and both of the resistance traits mapped in close proximity to S18_30104122 at position 98.1 cm. QTLs with significant LOD values were not identified when the standard model of interval mapping was performed with the Riesling 403 SNP set (S4A and S4B Fig).

### SNP validation and genetic mapping

The goal of mapping the *MJR1* locus was to identify a set of SNPs for predicting resistance to *M*. *javanica* ‘pt. 1103P’ for marker assisted selection. Therefore, 36 SNPs that mapped in close proximity to *MJR1* were selected for SNP genotyping using the Sequenom MassARRAY platform [51]. In this analysis, 6 SNPs could not be assessed including S18_30104122, which flanked *MJR1* (Fig 3), due to primer design constraints or failed PCR-genotype assays (S7 Table). After performing the SNP genotyping, 15 out of the remaining 30 SNPs genotyped were polymorphic and the genotypes matched with those predicted by TASSEL (Table 3). In addition, the major and minor allele frequencies identified by the Sequenom MassARRAY and TASSEL were equivalent. Of the 30 Sequenom MassARRAY genotyped SNPs, 11 SNPs were non-polymorphic and did not match the genotype predicted by TASSEL (Table 3). In addition, four polymorphic SNPs, S18_31481177, S18_33162606, S18_33959722 and S19_34060245 verified by Sequenom MassARRAY had the opposite genotype predicted by GBS (Table 3). For example, all heterozygous SNPs predicted by GBS were found to be homozygous by Sequenom MassARRAY and vice versa (data not shown). Taken together, the GBS pipeline accurately predicted the SNP genotype with a 50% success rate at the *MJR1* locus.

**Table 3:** Validation of 30 SNPs at the *MJR1* locus.

		Sequenom MassARRAY			TASSEL		
SNP ID	Chr	Genotype	MF	MAF	Genotype	MF	MAF
S18_26580875	18	GG/GA	0.72	0.28	GG/GA	0.72	0.28
S18_26558715	18	GG/GT	0.73	0.27	GG/GT	0.73	0.27
S18_27884817	18	CC/CT	0.73	0.27	CC/CT	0.73	0.27
S18_28372660	18	AA/AG	0.73	0.27	AA/AG	0.73	0.27
S18_30104225	18	CC/CG	0.72	0.28	CC/CG	0.72	0.28
S18_30226628	18	AA/AG	0.72	0.28	AA/AG	0.72	0.28
S18_30236024	18	GG/GA	0.72	0.28	GG/GA	0.72	0.28
S18_30711041	18	GG/GT	0.71	0.29	GG/GT	0.71	0.29
S18_30722375	18	GG/GA	0.71	0.29	GG/GA	0.71	0.29
S18_31160355	18	AA/AG	0.71	0.29	AA/AG	0.71	0.29
S18_31787750	18	GG/GA	0.72	0.28	GG/GA	0.72	0.28
S18_31886894	18	AA/AT	0.72	0.28	AA/AT	0.72	0.28
S18_32680428	18	AA/AG	0.72	0.28	AA/GA	0.72	0.28
S18_33070954	18	GG/GA	0.72	0.28	GG/GA	0.72	0.28
S18_33954011	18	AA/AG	0.71	0.29	AA/AG	0.71	0.29
S18_30381428	18	TT	1	0	TT/TC	0.71	0.29
S18_30515641	18	TT	1	0	TT/TA	0.71	0.29
S18_30764625	18	CC	1	0	CC/CT	0.71	0.29
S18_31059094	18	TT	1	0	TT/TG	0.71	0.29
S18_31158467	18	TT	1	0	TT/TA	0.78	0.22
S18_31164496	18	TT	1	0	TT/TC	0.71	0.29
S18_31196588	18	AA	1	0	AA/AT	0.71	0.29
S18_31547619	18	GG	1	0	GG/GA	0.75	0.25
S18_33070986	18	CC	1	0	CC/CT	0.72	0.28
S18_33276771	18	CC	1	0	CC/CT	0.71	0.29
S18_33876484	18	CC	1	0	CC/CT	0.71	0.29
S18_31481177	18	GG/GT	0.79	0.21	GG/GT	0.79	0.21
S18_33162606	18	CC/CA	0.79	0.21	CC/CA	0.79	0.21
S18_33959722	18	CC/CT	0.79	0.21	CC/CT	0.79	0.21
S18_34060245	18	AA/AG	0.78	0.22	AA/AG	0.78	0.22

SNPs validated by Sequenom MassARRAY for 65 of the F_1_ individuals were compared with results from the TASSEL GBS data. The marker name contains information regarding the position of the SNP in the PN40024 genome. SNPs were selected from position 26580875 to 34060245 for validation. Note: the first section of the table contains 15 SNPs in which genotypes determined by Sequenom MassARRAY matched results produced by the TASSEL GBS pipeline. In the two later sections, the genotype identified by Sequenom MassARRAY did not match with results from the TASSEL GBS pipeline. Chr = Chromosome; MF = Major Allele Frequency; MAF = Minor Allele Frequency.

**Fig 5:**
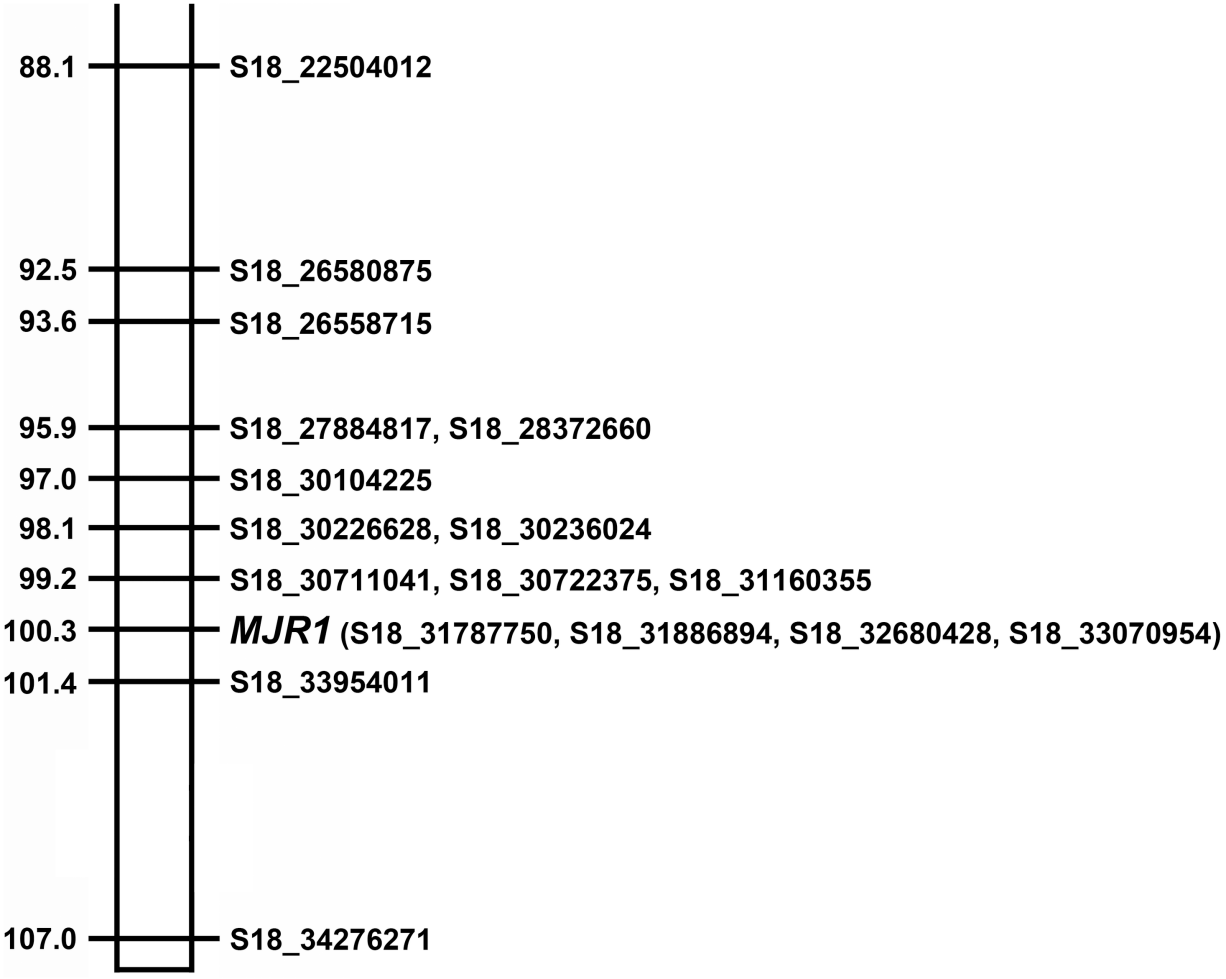
Linkage mapping of *MJR1* using validated SNPs. SNPs at the *MJR1* locus were validated using the Sequenom MassARRAY [51]. Non-polymorphic GBS-predicted SNPs and incorrectly genotyped SNPs were removed before mapping *MJR1* using the 380 SNP set. SNP ID is shown on the right and distance in cM on displayed on the left side of the linkage group.

To validate the previous genetic mapping results, a 380 SNP set was created, which included the 15 accurately genotyped SNPs at the *MJR1* locus, as well as the *MJR1* marker (see Materials and methods). When the 380 SNP was created, S18_30104122 and S18_33162606 were removed from the 367 SNP set, as the genotype for these markers could not be verified. Also, S18_27884817 was a validated SNP that was present in the 367 SNP set. Results from R/OneMap analysis showed that *MJR1* mapped to LG18 at position 100.3 cM (Fig 5). S18_31787750, S18_31886894, S18_32680428 and S18_33070954 cosegregated with *MJR1* at position 100.3 cM. *MJR1* was flanked by S18_30711041, S18_30722375 and S18_31160355 at 99.2 cM and S18_33954011 at 101.4 cM (Fig 5).

**Fig 6:**
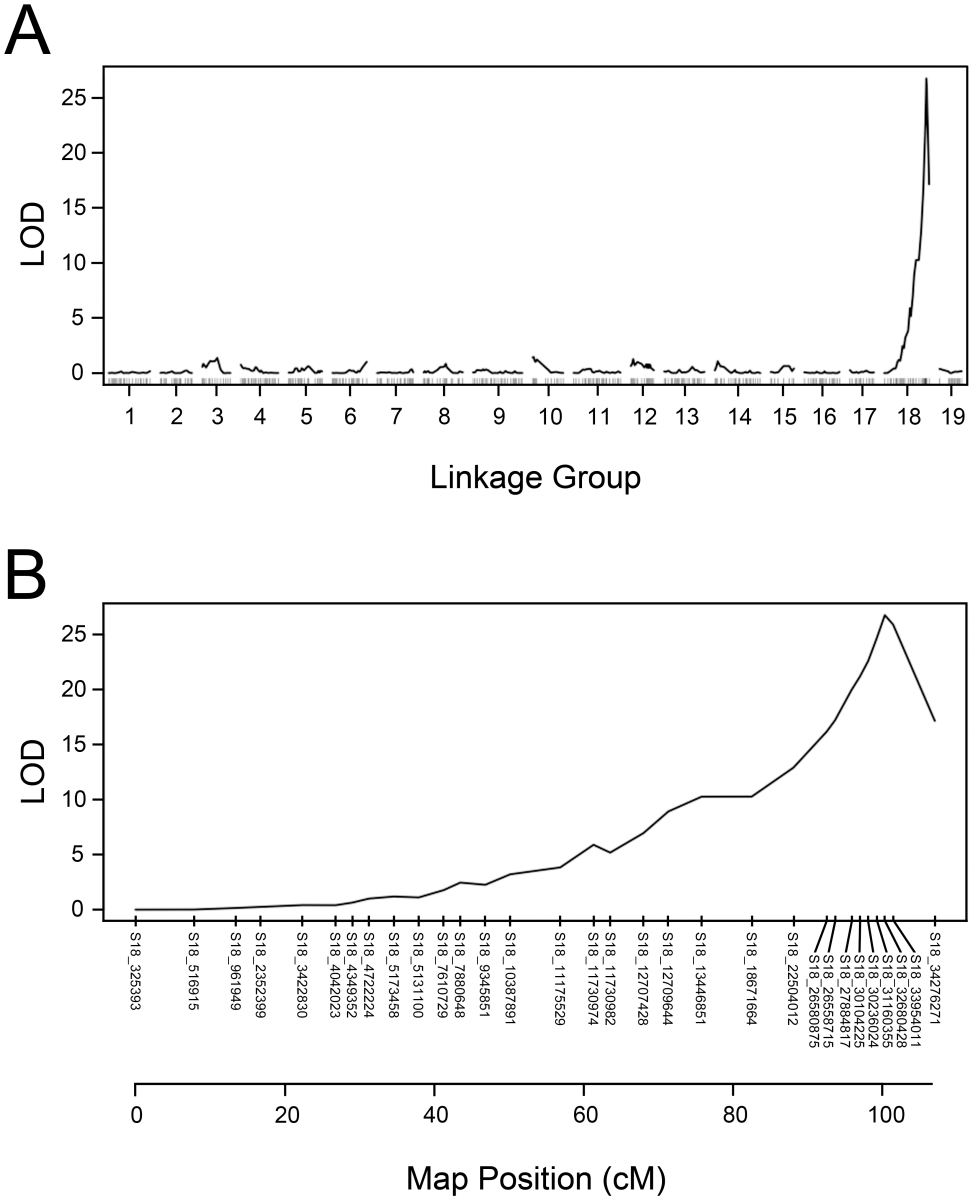
Binary model of interval mapping using validated SNPs at *MJR1* locus. Interval mapping was performed with the 372 SNP set, which contains eight validated markers (S18_26580875, S18_26558715, S18_27884817, S18_30104225, S18_30236024, S18_32680428, S18_33954011). (A) A single LOD maximum of 26.7 was detected on LG18. Linkage group number and LOD scores are displayed on the x-axis and y-axis, respectively. (B) A diagram of LG18 displayed the LOD maximum at 100.3 cM with markers. Markers and map position (cM) are displayed on the x-axis and LOD scores are shown on the y-axis. The threshold, as determined by 1000 permutations, was 4.81.

The binary and normal models of interval mapping were utilized to localize *MJR1* using the 372 SNP set, which contained 8 validated markers (see Materials and methods). Using the binary method, a LOD maximum of 26.7 (*p*-value = 0.0) was detected on LG18 and cosegregated with S18_32680428 at position 100.3 cM (Fig 6A and 6B). When using the EM/R or EM/RW traits with the normal model of interval mapping, a single QTL for *M*. *javanica* ‘pt 1103P’ resistance localized to LG18 at 100 cM with a LOD score of 8.56 (*p*-value = 0.001) or 7.75 (*p*-value = 0.003), respectively, which is in close proximity to S18_32680428 (S5A and S5B Fig). The LOD scores obtained by binary and standard methods of mapping were above the threshold values. Taken together, after validating SNPs, linkage and interval mapping support a model that the *MJR1* locus is located at ~100 cM on LG18.

### *Meloidogyne javanica* ‘pt 1103P’ induced resistance response in C2-50

**Fig 7:**
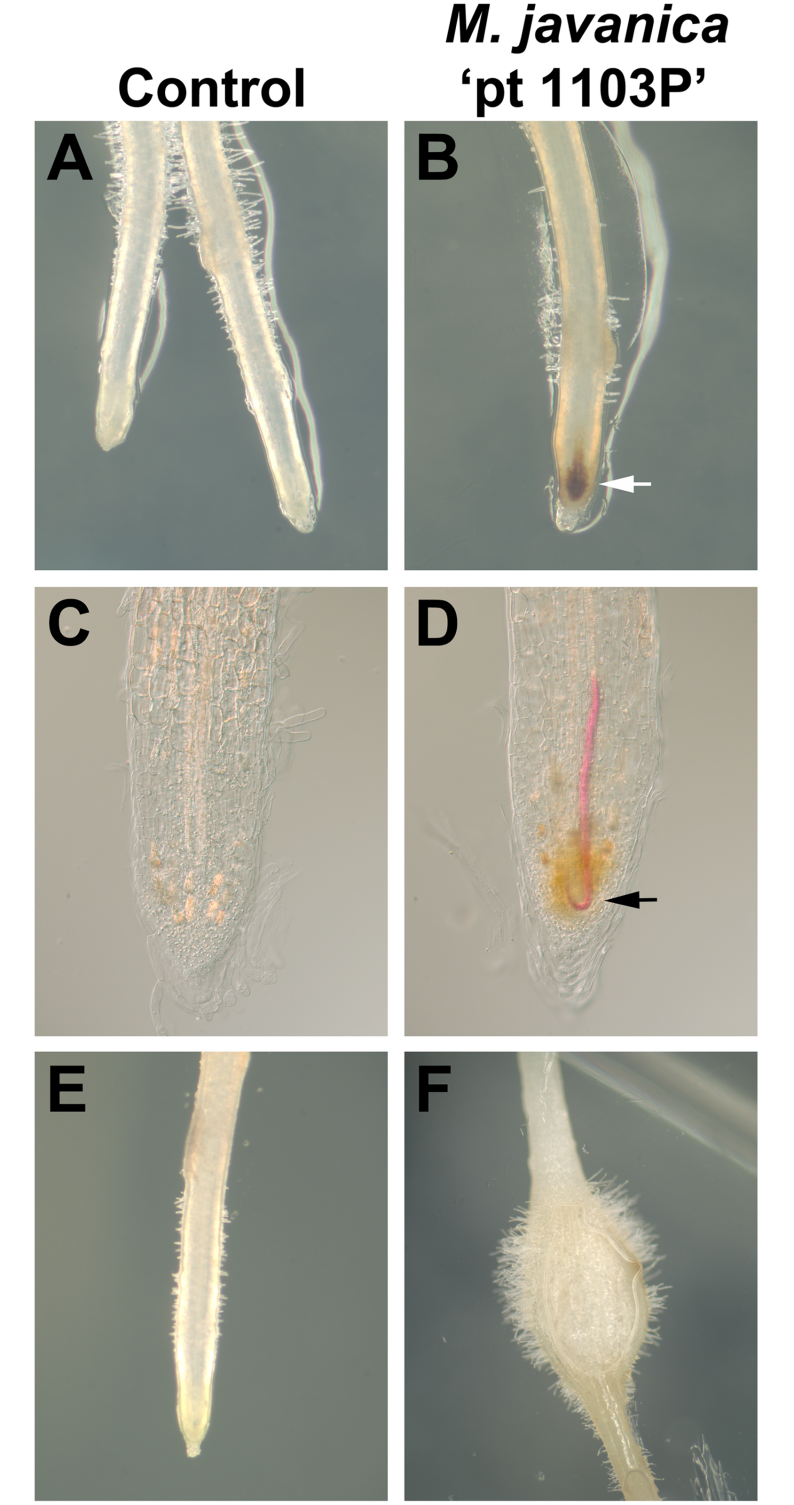
*Meloidogyne javanica* ‘pt 1103P’ induced cellular necrosis in the root meristem cells of C2-50. In vitro grown roots for (A-D) C2-50 and (E and F) Riesling. (A) C2-50 roots treated with sterile water. (B) C2-50 roots inoculated with *M*. *javanica* ‘pt. 1103P’. (B) White arrow points at *M*. *javanica* ‘pt 1103P’ induced cellular necrosis in root meristem. (C and D) C2-50 roots inoculated with *M*. *javanica* ‘pt 1103P’ and stained for nematodes using 10% bleach followed by acid fuchsin staining. (C) C2-50 roots with no cellular necrosis were not penetrated with a nematode(s). (D) C2-50 roots that displayed cell necrosis in root meristem had nematodes. Black arrow points at *M*. *javanica* ‘pt 1103P’ embedded in necrotic cells (Note: the 10% bleach treatment reduced the brown coloration of the necrotic cells in the root meristem). Riesling roots treated with (E) sterile water or (F) inoculated with *M*. *javanica* ‘pt 1103P’.

The biological basis of root knot nematode resistance was examined in C2-50 using an *in vitro* screening assay. In this experiment, C2-50 and Riesling roots were incubated with approximately 25 *M*. *javanica* ‘pt 1103P’ nematodes or a sterile water sample used as a control. C2-50 roots treated with the sterile water control did not undergo any visible signs of cellular necrosis, growth cessation or gall development (Fig 7A). Inoculation of C2-50 roots with *M*. *javanica* ‘pt 1103P’ induced cellular necrosis in the root meristem for 63% of the roots examined (Fig 7B, white arrow). The root meristem localized necrotic reaction was induced by 36–48 hours and resulted in a cessation of root growth. Gall formation was not induced in any of the C2-50 roots by this nematode. To determine if the cellular necrosis was due to presence of *M*. *javanica* ‘pt 1103P’, roots with and without cellular necrosis were stained for nematodes. In this procedure, roots were treated with 10% bleach to increase the transparency of the necrotic region in order to visualize the root knot nematode. Results showed that *M*. *javanica* ‘pt 1103P’ was absent from roots lacking cellular necrosis (Fig 7C). However, in roots with cellular necrosis, at least one *M*. *javanica* ‘pt 1103P’ was located in the root meristem (Fig 7D). Moreover, many of these root knot nematodes were curved like a hook, as if they were trapped while migrating through the root meristem and into the vascular cylinder (Fig 7D, black arrow). Results showed that Riesling roots treated with a sterile water sample were not altered in growth (Fig 7E). However, inoculation of Riesling roots with *M*. *javanica* ‘pt 1103P’ promoted gall formation in 80% of the roots (Fig 7F). In some cases, egg mass development was apparent after 21 days (data not shown).

The *M*. *javanica* ‘pt 1103P’ induced cell necrotic response was examined in 12 F_1_ C2-50 x Riesling individuals. In the *M*. *javanica* ‘pt 1103P’ screen performed in soil (pot trail), zero egg masses developed on the resistant F_1_ individuals, while a range of egg masses developed on the susceptible F_1_ individuals (Table 4). Using the in vitro screen, results showed that inoculation of *M*. *javanica* ‘pt 1103P’ induced cellular necrosis in the root meristem followed by root growth cessation in all three replicates for six F_1_ progeny that were resistant based on the *M*. *javanica* ‘pt 1103P’ pot trail (Table 4). Galling and egg mass production was not apparent in the roots isolated from these resistant F_1_ individuals. In contrast, gall formation was induced after the addition of *M*. *javanica* ‘pt 1103P’ in all three replicates for the six susceptible F_1_ individuals identified in the pot trial (Table 4). Cellular necrosis of the root meristem did not occur after the F_1_ susceptible individuals were inoculated with *M*. *javanica* ‘pt 1103P’. The addition of sterile water alone did not promote cellular necrosis in the root meristem or gall formation in any of the F_1_ individuals (data not shown).

**Table 4:** Comparison of *M*. *javanica* ‘pt 1103P’ screening assays.

	Pot Trial		In Vitro Screen	
F_1_ Individual	Egg Mass	Classification	Phenotype %	Classification
K2B_16_04_4	0.0	R	53% HR	R
K2B_16_04_7	0.0	R	80% HR	R
K2B_16_05_2	0.0	R	53% HR	R
K2B_16_07_5	0.0	R	47% HR	R
K2B_16_10_8	0.0	R	53% HR	R
K2B_16_12_6	0.0	R	60% HR	R
K2B_15_12_7	31.3	S	60% GD	S
K2B_15_14_4	44.0	S	60% GD	S
K2B_16_10_2	34.0	S	72% GD	S
K2B_16_12_3	33.0	S	53% GD	S
K2B_16_13_7	17.7	S	67% GD	S
K2B_16_13_8	13.7	S	53% GD	S

Egg Mass, Mean number of egg masses; R, resistant; S, susceptible;

Phenotype %, percent of roots with hypersensitive response (HR) or galls development (GD)

## Discussion

In this manuscript, an F_1_ mapping population derived from a C2-50 x Riesling cross was used to map the *M*. *javanica* ‘pt. 1103P’ resistance locus, *MJR1*. Results from the nematode-screening assay performed on the parents and F_1_ individuals indicated that *M*. *javanica* ‘pt. 1103P’ resistance is controlled by a single dominant allele, which is derived from the C2-50 female parent. Genetic studies indicate that resistance to *M*. *incognita* is conferred by a single dominant allele in the *V*. *champinii* rootstocks Harmony, Freedom, Dog Ridge and Ramsey [13]. At this time, it is not clear as to whether the location and identity of root knot nematode resistance locus and gene(s), respectively, is similar in C2-50 and the *V*. *champinii* derived rootstocks.

For protection against root knot nematode feeding, plants have evolved resistance mechanisms to reduce or eliminate reproduction. Experimental studies have shown that a subset of root knot nematode resistant plants exhibit a localized hypersensitive response (HR) induced during penetration, migration and/or gall development [2,57]. In grapevine, the spatial dynamics of HR was examined in five rootstocks with different species background using the virulent *M*. *arenaria* ‘pt Harmony’ for the nematode screening. Complete resistance with no egg mass development occurred in 10-23B (*V*. *doaniana*) [58] and Demko 10-17A (Edna x *V*. *simpsoni*) [59]. In 10-23B, HR was induced primarily in the vasculature [58], while cell necrosis occurred during penetration and early gall development in the root epidermis cells and vasculature, respectively, for Demko 10-17A [59]. A low level of *M*. *arenaria* ‘pt Harmony’ reproduction occurred in RS-3 (Ramsey (*V*. *champinii*) x Schwarzmann (*V*. *riparia* x *V*. *rupestris*)) [58], RS-9 (Ramsey (*V*. *champinii*) x Schwarzmann (*V*. *riparia* x *V*. *rupestris*)); [60] and 6-19B (*V*. *champinii* x GA-3, 4, 5) [59], which are all partially resistant to this root knot nematode. During root knot nematode penetration and early gall development, HR was induced in the epidermis and vasculature in RS-3 [58] and 6-19B [59]. In RS-9, root knot nematodes induced a HR in the epidermis and root tip during penetration and migration [60]. In contrast to the above studies, *M*. *javanica* ‘pt 1103P’ induced cell necrosis in the root meristem of the *V*. *cinerea* accession C2-50, which likely functions to inhibit root knot nematode migration, as well as perturb giant cell specification from vasculature initial cells. Given that the *M*. *javanica* ‘pt 1103P’ cellular necrotic induced phenotype was only apparent in the six resistant F_1_ genotypes this indicates that this mode of immunity is mediated by *MJR1*. The differences in the HR induced spatial patterns between C2-50, 10-23B, Demko 10-17A, RS-3 and RS-9 suggest that these genotypes possess different resistant genes, which is of interest for breeding rootstocks with durable resistance to root knot nematode.

Genetic and molecular studies have resulted in the identification and functional characterization of root knot nematode resistance genes in *Solanum peruvianum*, *S*. *arcanum* and *Prunus cerasifera* [61–63]. These root knot nematode genes encode classic resistant (R)-proteins containing a nucleotide binding (NB) and leucine-rich repeat domains (LRR). Candidate *R*-genes containing the NB and LRR domains have been identified in the *Vitis vinifera* genome [64–67]. An integrated genetic map derived from two grapevine crosses was used to map 82 *R*-genes and results showed that clusters of *R*-genes are located on chromosome 18 [67], which may overlap with the region corresponding to *MJR1*. Chromosome 18 also contains loci, which confer resistance to *Plasmopara viticola* [68–75] and *Erysiphe necator* [76,77] in North American *Vitis* and *Muscadinia* species. Results showing a high level of sequence similarity between *V*. *vinifera* and *M*. *rotundifolia* at the *Uncinula necator*/*Plasmopara viticola* resistant locus on chromosome 12 indicates that the group of *R*-genes at this locus are evolutionarily related in these two species [78]. Given that 16 *R*-genes, annotated as TMV resistance genes and 5 disease resistance genes implicated in plant immunity are located between S18_30711041 and S18_33954011 on chromosome 18 of PN40024 (data not shown), it may be possible that the resistant gene(s) at *MJR1* in C2-50 are evolutionarily related to the resistant genes at the corresponding locus in *V*. *vinifera*.

Recent studies have utilized next generation sequencing approaches to generate genetic maps for identifying favorable horticultural traits including powdery mildew resistance/susceptibility, flower sex and fruit quality traits in grapevine [22–29]. In our work, next generation sequencing followed by SNP calling/filtering further demonstrates the feasibility of using a GBS approach for SNP discovery, linkage map construction and mapping at least one Mendelian trait. However, validation of SNPs using Sequenom MassARRAY analysis indicated that the GBS pipeline results do not easily transfer with only 50% of the SNPs producing useful markers at the *MJR1* locus. The TASSEL-GBS pipeline was designed for marker discovery and SNP calling in inbred crops with the aim of identifying a large number of markers at low sequencing coverage [39]. While GBS [38], as well as restriction site associated DNA (RAD) Sequencing [79,80], are effective platforms for SNP discovery and genotyping in plants, little emphasis has been placed on validating SNPs [19–21,81]. Furthermore, in cases where SNPs have been validated, it is difficult to compare results due to differences in next generation sequencing approaches, quality or availability of a reference genome, depth coverage, length of sequence reads, alignment algorithms and SNP calling pipelines [82–87]. While the focus of our research was to identify SNPs linked to *MJR1* for marker assisted selection, results show that a validation step is necessary for assessing markers when the TASSEL-GBS pipeline is utilized for SNP discovery and genotyping in *Vitis spp*. and possibly other highly heterozygous plant species. To improve the accuracy of the SNP calling step, sequence tags >64 bp could be utilized for the alignment step. Alternatively, a RAD sequencing approach [79,80] combined with paired end sequencing has the potential to create longer sequence tags, which could significantly increase the accuracy of the alignment step [81,88].

Single SNP association analysis is used to map traits of interest in populations of unrelated individuals [89]. However, a recent study, which utilized single SNP association together with interval mapping, identified a QTL for powdery mildew susceptibility in a grapevine F_1_ mapping population [23]. Results showed that most SNPs with significant LOD values were highly associated with powdery mildew susceptibility. For *M*. *javanica* ‘pt 1103P’ resistance, SNPs that co-segregated and were tightly linked to *MJR1* also displayed significant association with nematode resistance. As indicated by Barba et al., 2014, single SNP association can easily be performed with a greater number of markers compared to standard genetic mapping software. Therefore, single SNP association may serve as a useful tool to narrow down a set of markers for genetic mapping.

## Conclusions

In summary, traditional breeding approaches to develop rootstocks with resistance to plant parasitic nematodes and other soil borne pests is costly, time consuming and dependent upon labor-intensive work including plant propagation and nematode screening. However, employing a marker assisted breeding approach with molecular markers that predict nematode resistance would be an efficient and cost-effective approach. Moreover, marker assisted selection will allow for stacking multiple root knot nematode resistant loci into a single genetic background for producing new rootstocks with durable resistance. Using genetic mapping approaches, results show that *MJR1* maps at approximately 100 cM on LG18 in *V*. *cinerea* C2-50. In addition, SNPs at the *MJR1* locus had high genome wide association. Based on the position of these SNPs from 30711041 to 33954011 on chromosome 18 of the PN40024 reference genome, the estimated size of the *MJR1* locus is 3.24 Mb. The resistance mechanism mediated by *MJR1* involves localized cell necrosis in the root meristem, which may function to impair nematode migration and giant cell formation. Validated SNPs that cosegregate and the flank the *MJR1* locus from 99.2 to 101.4 cM will serve as molecular markers for predicting root knot nematode resistance in *V*. *cinerea* C2-50 for rootstock development.

## Supporting information

S1 Fig Distribution of *M*. *javanica* ‘pt 1103P’ phenotype data. The (A) average number of egg masses per root system and (B) average number of egg masses per root weight (g) was determined by screening three propagated cuttings for each F_1_ individual. The standard deviation is displayed in the solid black lines. Note: egg mass development was detected on all three replicates for the susceptible F_1_ individuals.
(TIF)

S2 Fig Interval mapping for *M*. *javanica* ‘pt 1103P’ resistance in Riesling. The binary model of mapping was used to localize *M*. *javanica* ‘pt 1103P’ resistance using the Riesling 403 SNP set. No significant maximum LOD scores that were above the LOD threshold of 3.25 were detected in Riesling SNP set. for *M*. *javanica* ‘pt 1103P’ resistance. The LOD threshold was determined using 1000 permutations with alpha = 0.05.
(TIF)

S3 Fig Single QTL analysis for *M*. *javanica* ‘pt 1103P’ resistance with the C2-50 367 SNP set. The standard model of interval mapping identified a single QTL on LG18 for (A) egg mass per root system at 97 cM with a LOD score equal to 7.27, which was above the LOD threshold value equal to 5.34. A single QTL on LG18 for (B) egg mass per root weight was identified at 98 cM with a LOD score equal to 6.8, which was above the LOD threshold value equal to 4.77.
(TIF)

S4 Fig QTLs for *M*. *javanica* ‘pt 1103P’ resistance were not detected in the Riesling 403 SNP set. The standard model of interval mapping was used to identify a single QTL in the Riesling 403 SNP set for (A) egg mass per root system (EM/R) and (B) egg mass per root weight (EM/RW). The LOD threshold values for EM/R and RM/RW were 6.14 and 4.80, respectively, with alpha = 0.05.
(TIF)

S5 Fig Single QTL analysis for *M*. *javanica* ‘pt 1103P’ resistance with the C2-50 372 SNP set. The 372 C2-50 SNP set contains eight of the fifteen validated markers. The standard model of interval mapping identified a single QTL on LG18 at 100 cM using (A) egg mass per root system (EM/R) and (B) egg mass per root weight (EM/RW) with LOD values equal to 8.56 and 7.75. These LOD scores are above the threshold values equal to 5.54 and 4.81 for EM/R and EM/RW, respectively, with alpha = 0.05.
(TIF)

S1 Table Single SNP association using GLM for the C2-50 3974 SNP set. Raw *p*-values obtained from GLM were adjusted (Adj.) using Bonferroni and Benjamini-Hockberg (Ben Hoch). Note: the name of the SNP contains chromosome and position information. For example, S18_31787750 is located on chromosome 18 at position 31787750 in the PN40024 reference genome.
(XLSX)

S2 Table Single SNP association using MLM for the 3974 C2-50 SNP set. Raw *p*-values obtained from MLM were adjusted (Adj.) using Bonferroni and Benjamini-Hockberg (Ben Hoch). Note: the name of the SNP contains chromosome and position information. For example, S18_31787750 is located on chromosome 18 at position 31787750 in the PN40024 reference genome.
(XLSX)

S3 Table Single SNP association using GLM for the Riesling 2973 SNP set. Raw *p*-values obtained from GLM were adjusted (Adj.) using Bonferroni and Benjamini-Hockberg (Ben Hoch). Note: the name of the SNP contains chromosome and position information. For example, S18_31114948 is located on chromosome 18 at position 31114948 in the PN40024 reference genome.
(XLSX)

S4 Table Single SNP association using MLM for the Riesling 2973 SNP set. Raw *p*-values obtained from MLM were adjusted (Adj.) using Bonferroni and Benjamini-Hockberg (Ben Hoch). Note: the name of the SNP contains chromosome and position information. For example, S18_31114948 is located on chromosome 18 at position 31114948 in the PN40024 reference genome.
(XLSX)

S5 Table C2-50 genetic map. The genetic map was constructed with 367 SNPs. This table contains the ordered markers with position information (cM) for each of the 19 linkage groups. Linkage group size is determined by the position (cM) of the last marker in each linkage group. The total size for the C2-50 genetic map is 1587.3 cM.
(XLSX)

S6 Table Riesling genetic map. Four hundred and three SNPs were used to construct the Riesling genetic map. The ordered markers and position (cM) for each of the 19 linkage groups is displayed. Linkage group size is determined by the position (cM) of the last marker in each linkage group. The size of the Riesling genetic map is 1706.4 cM.
(XLSX)

S7 Table The list of SNPs that could not be evaluated by Sequenome MassARRAY analysis. (XLSX)
