# Supplementary figures and images for: SNP markers tightly linked to root knot nematode resistance in grapevine (Vitis cinerea) identified by a genotyping-by-sequencing approach followed by Sequenom MassARRAY validation
Source: PLoS One. 2018 Feb 20;13(2):e0193121. doi: 10.1371/journal.pone.0193121 (PMC5819801; doi:10.1371/journal.pone.0193121)

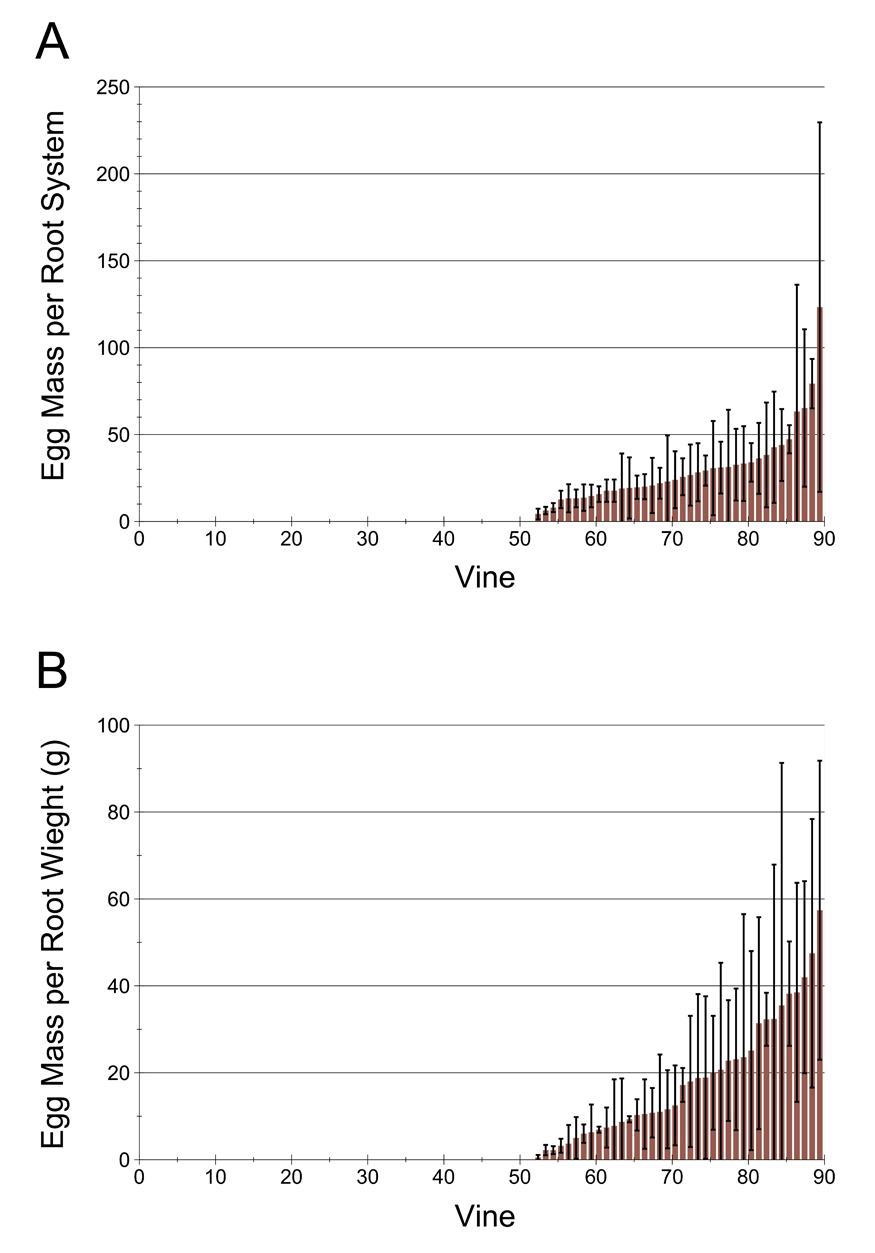

Supplement: S1 Fig — The (A) average number of egg masses per root system and (B) average number of egg masses per root weight (g) was determined by screening three propagated cuttings for each F1 individual. The standard deviation is displayed in the solid black lines. Note: egg mass development was detected on all three replicates for the susceptible F1 individuals. (TIF) [file pone.0193121.s001.tif]

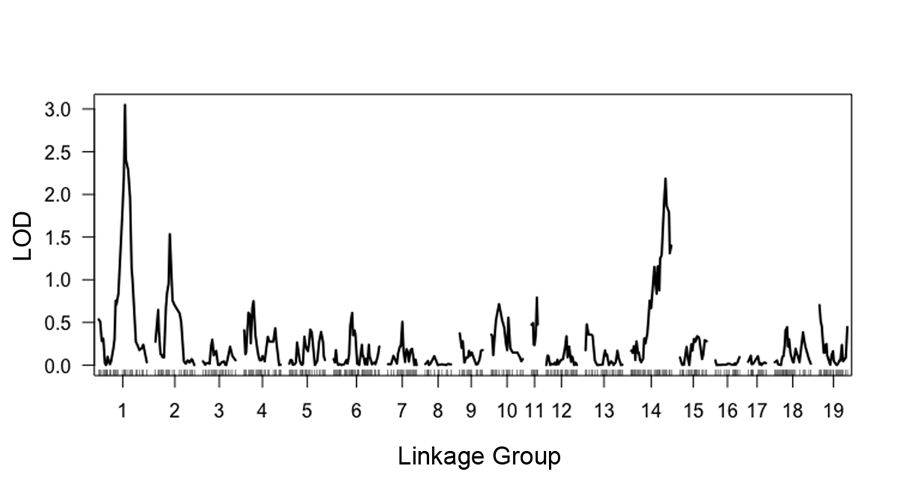

Supplement: S2 Fig — The binary model of mapping was used to localize M. javanica ‘pt 1103P’ resistance using the Riesling 403 SNP set. No significant maximum LOD scores that were above the LOD threshold of 3.25 were detected in Riesling SNP set. for M. javanica ‘pt 1103P’ resistance. The LOD threshold was determined using 1000 permutations with alpha = 0.05. (TIF) [file pone.0193121.s002.tif]

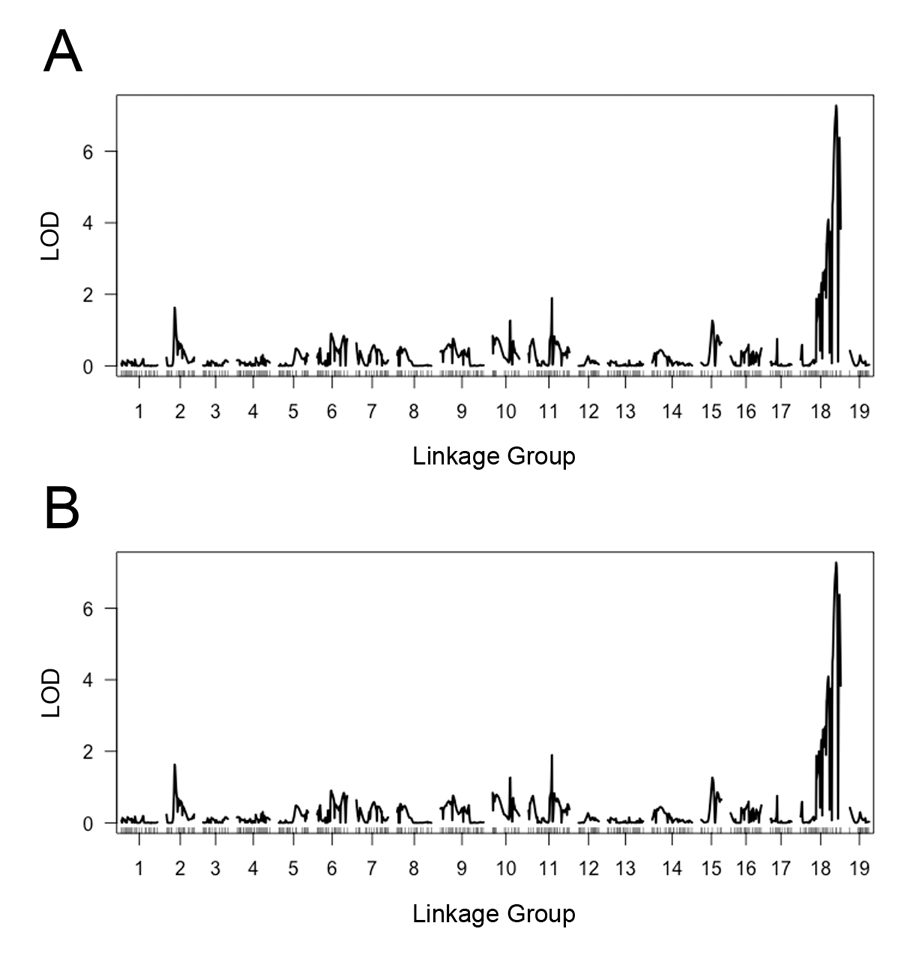

Supplement: S3 Fig — The standard model of interval mapping identified a single QTL on LG18 for (A) egg mass per root system at 97 cM with a LOD score equal to 7.27, which was above the LOD threshold value equal to 5.34. A single QTL on LG18 for (B) egg mass per root weight was identified at 98 cM with a LOD score equal to 6.8, which was above the LOD threshold value equal to 4.77. (TIF) [file pone.0193121.s003.tif]

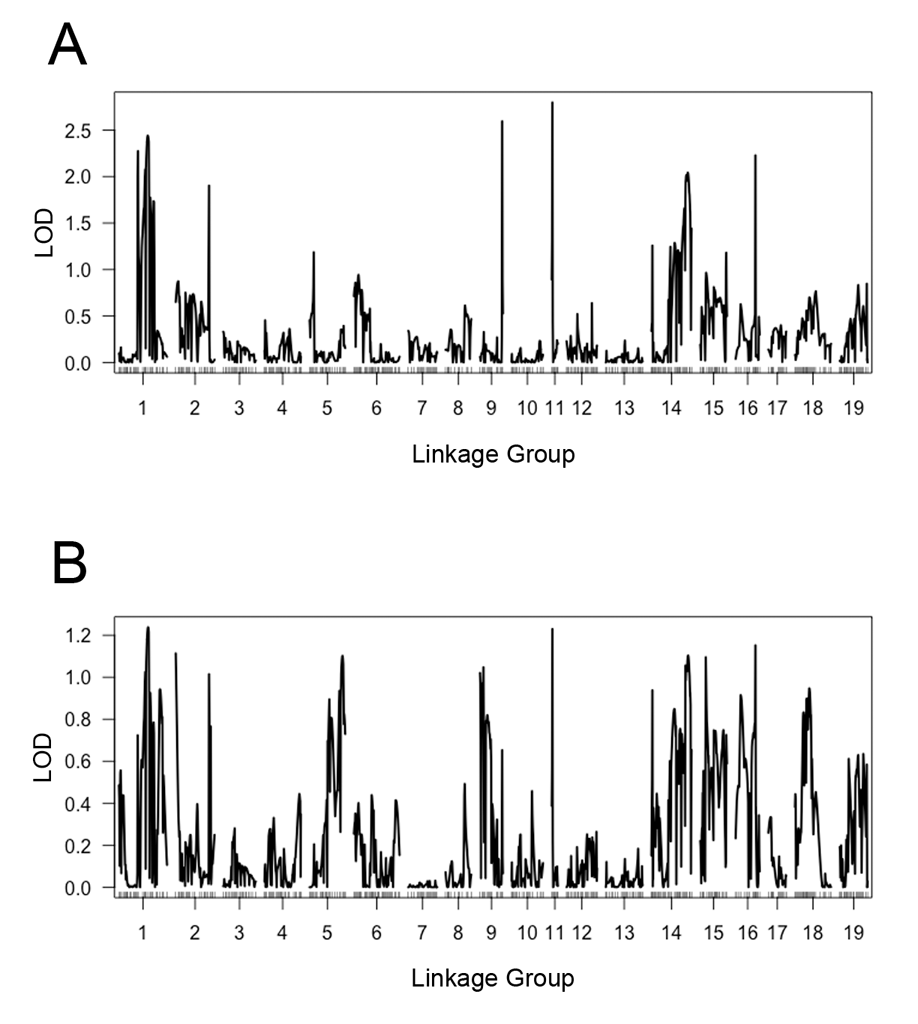

Supplement: S4 Fig — The standard model of interval mapping was used to identify a single QTL in the Riesling 403 SNP set for (A) egg mass per root system (EM/R) and (B) egg mass per root weight (EM/RW). The LOD threshold values for EM/R and RM/RW were 6.14 and 4.80, respectively, with alpha = 0.05. (TIF) [file pone.0193121.s004.tif]

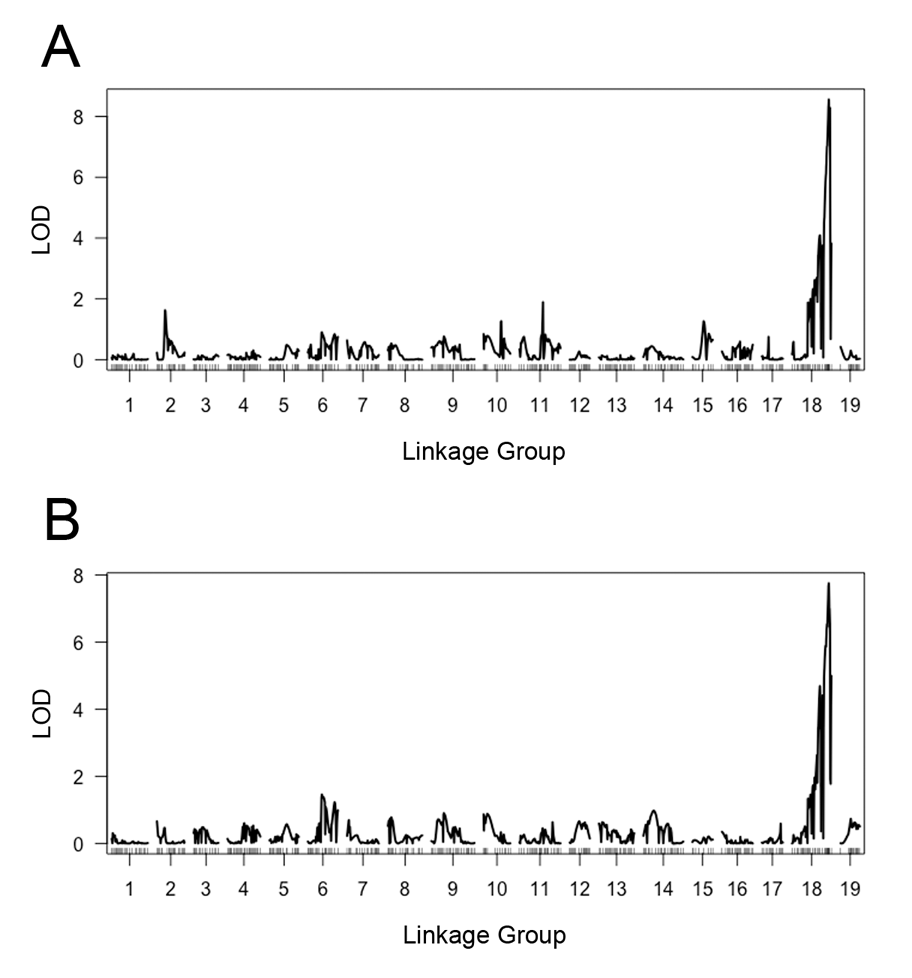

Supplement: S5 Fig — The 372 C2-50 SNP set contains eight of the fifteen validated markers. The standard model of interval mapping identified a single QTL on LG18 at 100 cM using (A) egg mass per root system (EM/R) and (B) egg mass per root weight (EM/RW) with LOD values equal to 8.56 and 7.75. These LOD scores are above the threshold values equal to 5.54 and 4.81 for EM/R and EM/RW, respectively, with alpha = 0.05. (TIF) [file pone.0193121.s005.tif]
